# Chronic intermittent ethanol promotes ventral subiculum hyperexcitability via increases in extrinsic basolateral amygdala input and local network activity

**DOI:** 10.1038/s41598-021-87899-0

**Published:** 2021-04-22

**Authors:** Eva C. Bach, Sarah E. Ewin, Alexandra D. Baldassaro, Hannah N. Carlson, Jeffrey L. Weiner

**Affiliations:** Department of Physiology and Pharmacology, Wake Forest Baptist School of Medicine, PTCRC 212, 115 South Chestnut Avenue, Winston-Salem, NC 27284 USA

**Keywords:** Neural circuits, Neuroscience, Addiction

## Abstract

The hippocampus, particularly its ventral domain, can promote negative affective states (i.e. stress and anxiety) that play an integral role in the development and persistence of alcohol use disorder (AUD). The ventral hippocampus (vHC) receives strong excitatory input from the basolateral amygdala (BLA) and the BLA-vHC projection bidirectionally modulates anxiety-like behaviors. However, no studies have examined the effects of chronic alcohol on the BLA-vHC circuit. In the present study, we used ex vivo electrophysiology in conjunction with optogenetic approaches to examine the effects of chronic intermittent ethanol exposure (CIE), a well-established rodent model of AUD, on the BLA-vHC projection and putative intrinsic vHC synaptic plasticity. We discovered prominent BLA innervation in the subicular region of the vHC (vSub). CIE led to an overall increase in the excitatory/inhibitory balance, an increase in AMPA/NMDA ratios but no change in paired-pulse ratios, consistent with a postsynaptic increase in excitability in the BLA-vSub circuit. CIE treatment also led to an increase in intrinsic network excitability in the vSub. Overall, our findings suggest a hyperexcitable state in BLA-vSub specific inputs as well as intrinsic inputs to vSub pyramidal neurons which may contribute to the negative affective behaviors associated with CIE.

## Introduction

Alcohol use disorder (AUD) is characterized by the compulsive consumption of alcohol despite negative consequences associated with this behavior. AUD is known to lead to cognitive impairments, memory deficits and the development of a negative affective state that manifests during periods of abstinence^1–3^. Although all of these alterations contribute to the etiology of AUD, our understanding of the neural circuits that drive these maladaptive phenotypes remains incomplete.

The hippocampus is critically involved in memory processes and the modulation of affective states. There are functional differences along the dorsoventral (human anteroposterior) axis of this brain region. Despite general functional overlap it is predominantly the ventral/anterior hippocampus (vHC) that is known to play an important role in discriminating the emotional context of hippocampal functions. Notably, the vHC modulates negative affective states (i.e. stress and anxiety)^4,5^, which play a causative role in the development and persistence of AUD. Evidence from human imaging studies showing reductions in hippocampal volume in individuals with AUD or affective disorders support the comorbid nature of these conditions^6–8^. Additionally, structural changes of the hippocampus correlate with memory deficit severities in AUD patients^3,6,9^, pointing to the hippocampus as a uniquely positioned neuronal region involved in multiple pathophysiological consequences associated with alcohol dependence.

Animal models of AUD have provided a window into the progression of synaptic plasticity events that are presumed to play a causative role in driving anatomical changes and maladaptive cognitive and behavioral outcomes associated with this disease^10^. Animals experiencing withdrawal from chronic intermittent ethanol exposure (CIE), an animal model of AUD^11,12^, show deficits in hippocampus-dependent memory function as well as increased anxiety-like behavior^13,14^. Neurophysiologically, the hippocampus, and particularly the ventral hippocampus (vHC), exhibits hyperexcitability following CIE^14,15^. Given the role of the vHC in modulating affective states, this finding fits with the notion that affective disorders and AUD are comorbid conditions that share circuit plasticity mechanisms^16,17^. Indeed chronic stress increases vHC excitability in a manner similar to chronic alcohol exposure^14,18,19^.

Afferent inputs and efferent projections link the vHC to other brain regions implicated in driving affective states and the pathophysiology of AUD^20–23^. Notably, the vHC is reciprocally connected with the basolateral amygdala (BLA). Hyperexcitability of BLA principal neurons plays a causal role in the anxiogenic phenotype promoted by CIE and BLA-vHC circuitry regulates anxiety-like phenotypes^21,24^. Despite this compelling evidence that CIE promotes hyperexcitability in both the BLA and vHC, and that activation of the BLA-vHC projection modulates affective behaviors, the effects of CIE on this circuit have not been examined.

In the present study we explored the role of the BLA-vHC pathway in driving plasticity of the vHC during withdrawal from CIE. First, using optogenetic approaches, we optically stimulated BLA inputs and recorded from neurons in the ventral subiculum (vSub) of the vHC. This approach revealed an increase in BLA-driven excitatory and inhibitory neurotransmission with the overall input-balance (excitatory/inhibitory ratio) being shifted to facilitate a hyperexcitable state in vSub neurons of CIE-treated rats. We also explored local network activity, which similarly revealed an increase in excitability, although this plasticity appeared to be mediated, at least in part, by a distinct mechanism from that observed at BLA-vSub synapses.

## Results

### The excitatory to inhibitory balance is disrupted in CIE treated rats to facilitate hyperexcitability of vSub neurons

**Figure 1:**
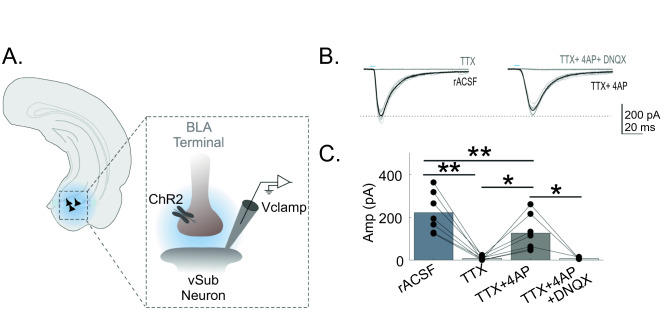
vSub neurons receive monosynaptic input from the BLA. (**A**) Schematic representation of recording configuration. Schematic line drawing of the hippocampus is adapted from Paxinos and Watson (2005) with permission^26^. (**B**) Representative oEPSCs in rACSF (black traces), during bath application of TTX (gray traces) on the right and bath application of TTX and 4AP (black traces) and TTX, 4AP and DNQX (gray traces). In representative traces, individual oEPSCs are shown by thin lines while the average response is illustrated by a thicker overlaid line. Blue bars above figures illustrate onset and duration of optical stimulation. (**C**) Optically- evoked input is abolished by TTX, reinstated by TTX + 4AP, and can be blocked by DNQX. *p < 0.05 and **p < 0.01 and errors are reported as ± SEM.

To study the BLA-specific inputs to vHC neurons we transfected the BLA with a virus encoding the expression of ChR2. After allowing for the expression and trafficking of ChR2 (5 weeks), animals were exposed to either air or CIE for 10–12 days. Following 24-h of withdrawal we conducted whole-cell electrophysiology to explore CIE-associated changes in synaptic conductance. Throughout our initial set of recordings we found pyramidal neurons of the vSub to most reliably receive functional input from the BLA and thus focused our studies on this region of the vHC. We first sought to confirm that these projections represent monosynaptic BLA inputs to the vSub. To establish the monosynaptic nature of these BLA inputs we optically stimulated BLA terminals to record oEPSCs at − 70 mV in slices prepared from air and CIE rats (Fig. 1A). We subsequently applied the sodium channel blocker TTX which blocked oEPSCs (221.9 ± 13.5 pA before TTX, 8.5 ± 1.3 pA with TTX, p = 0.0019, n = 7, Repeated measures ANOVA, Fig. 1B,C). The co-application of the broad-spectrum potassium channel blocker 4AP was able to restore currents blocked by TTX (125.9 ± 12.0 pA, p = 0.0216, n = 7, Repeated measures ANOVA, Fig. 1B,C). In a small subset of recordings, we applied the AMPA receptor antagonist DNQX, DNQX abolished the 4-AP recovered oEPSCs (8.0 ± 3.0 pA, n = 3, p = 0.0440, Repeated measures ANOVA). These results confirmed that the BLA makes monosynaptic glutamatergic projections onto vSub neurons. To determine whether vSub neurons of CIE animals experience changes in synaptic strength, we first compared monosynaptic BLA inputs between air and CIE groups. To accomplish this aim we optically stimulated BLA inputs, using identical optical stimulation parameters (duration/intensity), In these recordings we did not isolate monosynaptic inputs using TTX and 4AP. We considered inputs to be monosynaptic when responses showed a peak amplitude with a short response onset latency (< 5 ms following optical stimulation) with low jitter, as well as a linear rise time. There were a few instances where responses were included in this analysis despite showing additional disynaptic activity, as long as the first short latency peak responses could be clearly dissociated from any other recurrent inputs^25^.

**Figure 2:**
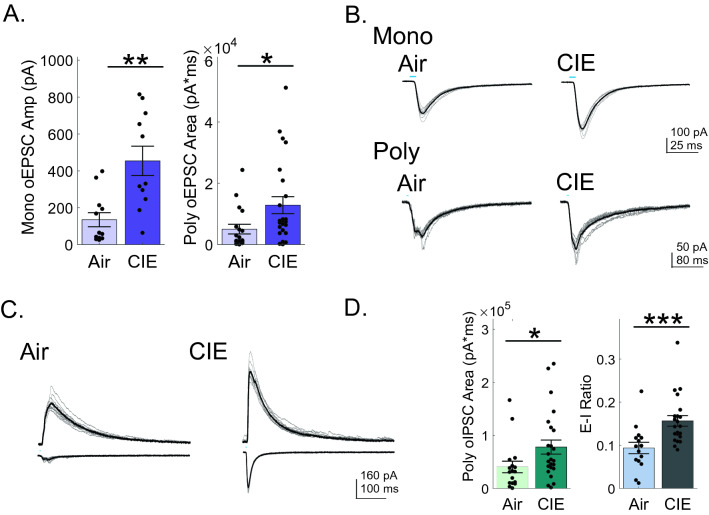
CIE shifts the excitatory/inhibitory balance in the BLA-vSub pathway toward a hyperexcitable state (**A**) Monosynaptic oEPSC amplitudes (left graph) and polysynaptic oEPSC areas (right graph) are increased in vSub neurons of CIE rats. (**B**) Representative monosynaptic (upper traces) and polysynaptic (lower traces) traces from a vSub neuron of Air (left column) and CIE (right column) rats. (**C**) Representative traces of oIPSCs (upper traces) and oEPSCs (lower traces) recorded from the same neuron. (**D**) Polysynaptic oIPSC areas are increased in vSub neurons of CIE rats (right panel) but this increase is smaller than the relative increase in oEPSC areas, resulting in an overall greater E/I ratio (right panel). In representative traces, individual inputs are shown by thin lines while the average response amplitude is illustrated by a thicker overlaid line. Blue bars above figures illustrate onset and duration of optical stimulation. *p < 0.05, ** p < 0.01 and ***p < 0.001 and errors are reported as ± SEM.

The amplitudes of these presumptive monosynaptic inputs to vSub neurons were significantly greater in CIE (454.2 ± 76.1 pA, n = 11, Fig. 2A,B) than air (135 ± 38.4 pA, n = 12, Fig. 2A,B) exposed rats (Mann–Whitney test, p = 0.0147). In a substantial subset of neurons, short latency optically-evoked responses were composed of multiple, indissociable peak responses indicative of di/polysynaptic recurrent excitatory activity. This finding was not surprising given the established abundance of recurrent connections within subicular pyramidal neurons (Bohm et al. 2015; Wee et al.^2020^). To explore this recurrent excitatory activity, we compared the total oEPSC input area between our animal groups. CIE (12,884.1 ± 3034.4 pA ms, n = 24, Fig. 2A,B) rats had significantly greater total excitatory neurotransmission than air (5064.5 ± 1618.4 pA ms n = 17, Fig. 2A,B) rats (Mann–Whitney test, p = 0.0178). Pyramidal neurons are also heavily interconnected with GABAergic interneurons (Bohm 2015), which led us to extend our analysis to exploring optically-evoked inhibitory neurotransmission. The overall optically-driven BLA inhibitory input of vSub neurons was also significantly greater in recordings from CIE (77,780.6 ± 16,841.5 pA ms, n = 24, Fig. 2C,D) vs. air (40,277.4 ± 10,834.0 pA ms, n = 17, Fig. 2C,D) rats (Mann–Whitney test, p = 0.0304). The increase in both excitatory and inhibitory neurotransmission raised the question whether the balance between excitatory and inhibitory input of CIE animals was disrupted in BLA driven inputs. To explore this question, we compared the input area of excitatory and inhibitory neurotransmission (E–I ratio) when both measures could be obtained in the same neuron. CIE rats had a significantly greater E–I ratio (0.16 ± 0.01, n = 23, Fig. 2C,D) compared to their air (0.09 ± 0.01, n = 15, Fig. 2C,D) counterparts (Mann–Whitney test, p = 0.0007). Taken together these findings suggest that, despite the increase of both inhibitory and excitatory neurotransmission, the overall balance is shifted to facilitate a BLA-driven hyperexcitable state in vSub neurons.

### Presynaptic release probability remains unaffected by CIE treatment

**Figure 3:**
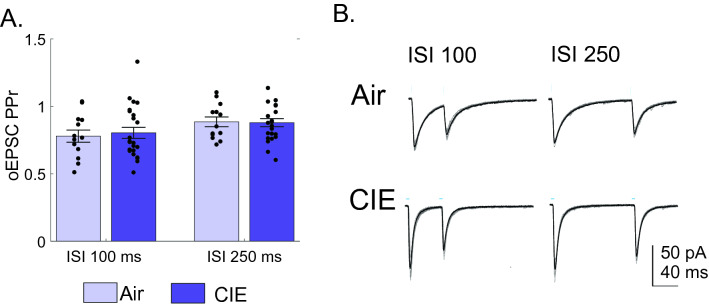
Presynaptic release probability of BLA-vSub oEPSCs is not altered in CIE rats. (**A**) PPr of oEPSCs at 100 and 250 ms ISI are unchanged in vSub neurons of Air and CIE rats. (**B**) Representative traces of oEPSC evoked at 100 (left column) and 250 (right column) ms ISIs in vSub neurons of Air (upper traces) and CIE (lower traces) rats. In representative traces individual inputs are shown by thin lines while the average response amplitude is illustrated by a thicker overlaid line. Blue bars above figures illustrate onset and duration of optical stimulation. Errors are reported as ± SEM.

To begin to assess the synaptic locus impacted by CIE, we used paired-pulse ratios (PPr) to assess presynaptic glutamate release probability at BLA-vSub synapses. The stimulation parameters were adjusted to evoke monosynaptic paired stimuli (100 and 250 ms ISI) and the Peak 2/Peak 1 ratio was calculated. We did not observe a change in the PPrs at either ISI between CIE (0.78 ± 0.04 at 100 ms ISI, n = 14; 0.88 ± 0.03 at 250 ms ISI, n = 14, Fig. 3A,B) and air (0.81 ± 0.04 at 100 ms ISI, n = 24; 0.88 ± 0.03 at 250 ms ISI, n = 25, Fig. 3A,B) rats (100 ms ISI p = 0.5242; 250 ms ISI p = 0.8913; two-tailed Student’s T-test). This finding suggests that the CIE treatment predominantly drives BLA-driven postsynaptic plasticity in vSub neurons.

### Postsynaptic plasticity is driven by an increase in AMPA-mediated EPSCs resulting in an elevated AMPA–NMDA ratio

**Figure 4:**
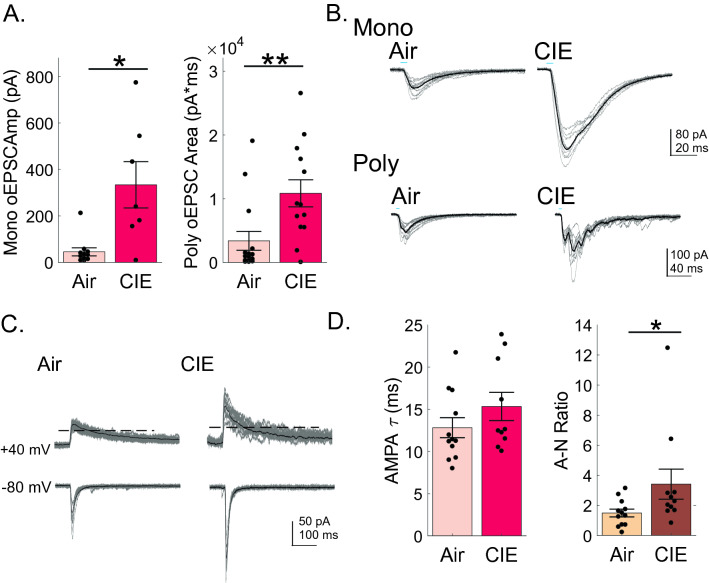
AMPA-mediated oEPSCs and AMPA/NMDA ratios are increased in vSub neurons of CIE rats. (**A**) Evoked monosynaptic amplitudes (left graph) and polysynaptic areas (right graph) of AMPA-mediated oEPSCs of vSub neurons in the presence of PTX are increased in vSub neurons of CIE rats. (**B**) Representative traces of monosynaptic (upper traces) and polysynaptic (lower traces) AMPA-mediated oEPSCs shown in (**A**) of Air (left column) and CIE (right column) rats. (**C**) oEPSCs recorded in the presence of PTX at − 80 mV (lower traces) to isolate AMPA-mediated EPSC amplitude and at + 40 mV (upper traces) to establish the NMDA-mediated amplitude within the same vSub neuron. (**D**) CIE rats have an increased AMPA/NMDA ratio (right graph) in CIE rats that cannot be explained by a change in AMPA tau (left graph). In representative traces, individual inputs are shown by thin lines while the average response amplitude is illustrated by a thicker overlaid line. Blue bars above figures illustrate onset and duration of optical stimulation. *p < 0.05 and ** p < 0.01 and errors are reported as ± SEM.

To further characterize CIE-mediated plasticity of BLA-vSub excitatory neurotransmission, we isolated AMPAR-mediated oEPSCs by blocking GABA_A_-receptor mediated inhibitory inputs (PTX application) and recorded at − 80 mV, where NMDARs remain largely under voltage- -dependent block (Mayer et al. 1984). ^31^.Again, we first, compared the monosynaptic BLA-driven input between our experimental groups. We found that monosynaptic AMPAR-mediated oEPSC amplitudes were significantly increased in recordings from CIE (333.9 ± 99.7 pA, n = 7, Fig. 4A,B) compared to Air (45.4 ± 17.4 pA, n = 11, Fig. 4A,B) rats (Mann–Whitney test, p = 0.0114). Qualitatively, we saw an even greater propensity of inputs to be contaminated by indissociable, di/polysynaptic activity which we attribute to increased network excitability. When we compared the total optically-driven activity of vSub neurons, using the area of AMPAR oEPSCs, we saw significantly greater activity in CIE (10,843.8 ± 2899.8 pA ms, n = 13, Fig. 4A,B) than Air (3375.9 ± 2899.8 pA ms, n = 15, Fig. 4A,B) rats (Mann–Whitney test, p = 0.0066). Postsynaptic glutamate plasticity is commonly associated with a change in the ratio between AMPAR- and NMDAR-mediated conductances^27,28^ and changes in AMPA–NMDA ratios have been seen in many brain regions associated with the pathophysiology of addiction^28,29^. To establish whether CIE altered the AMPA–NMDA ratio, we adjusted the stimulation strength to evoke monosynaptic AMPAR-mediated oEPSCs at − 80 mV and NMDAR -mediated EPSCs at + 40 mV. To determine the AMPA–NMDA ratio, we took the ratio of AMPAR- and NMDAR- mediated monosynaptic EPSC amplitudes when both could be obtained in the same neuron. We found the AMPA–NMDA ratio to be significantly greater in CIE (2.44 ± 0.51, n = 10, Fig. 4C,D) than air (1.38 ± 0.25, n = 11, Fig. 4C,D) rats (Mann–Whitney test, p = 0.0447). This increase in AMPAR-mediated EPSC input and a change in the AMPA–NMDA ratio reinforces our conclusion that postsynaptic plasticity facilitates hyperexcitability of BLA-vSub synapses as a consequence of CIE treatment.

### CIE increases excitatory, but not inhibitory, spontaneous neurotransmission in vSub neurons

**Figure 5:**
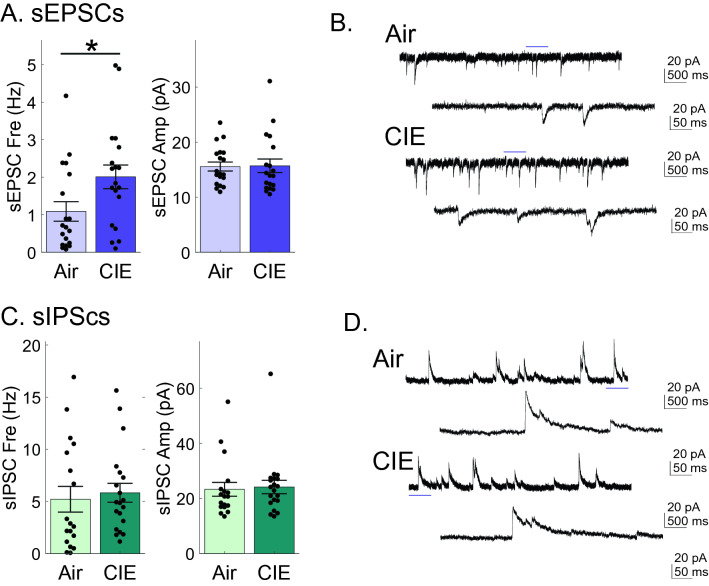
CIE-treated rats show an increase in spontaneous synaptic release of glutamate, but not GABA. (**A**) CIE rats have an increased sEPSC frequency (left graph), but not amplitude (right graph). (**B**) Representative sEPSC traces recorded from vSub neurons of Air (top two traces) and CIE (lower two traces) rats. (**C**) CIE rats do not show a change in frequency (left graph) or amplitude (right graph) of sIPSCs. (**D**) Representative sIPSC traces recorded from vSub neurons of Air (top two traces) and CIE (lower two traces) rats. Blue line above upper traces indicates zoomed in section illustrated in lower traces. *p < 0.05 and errors are reported as ± SEM.

**Figure 6:**
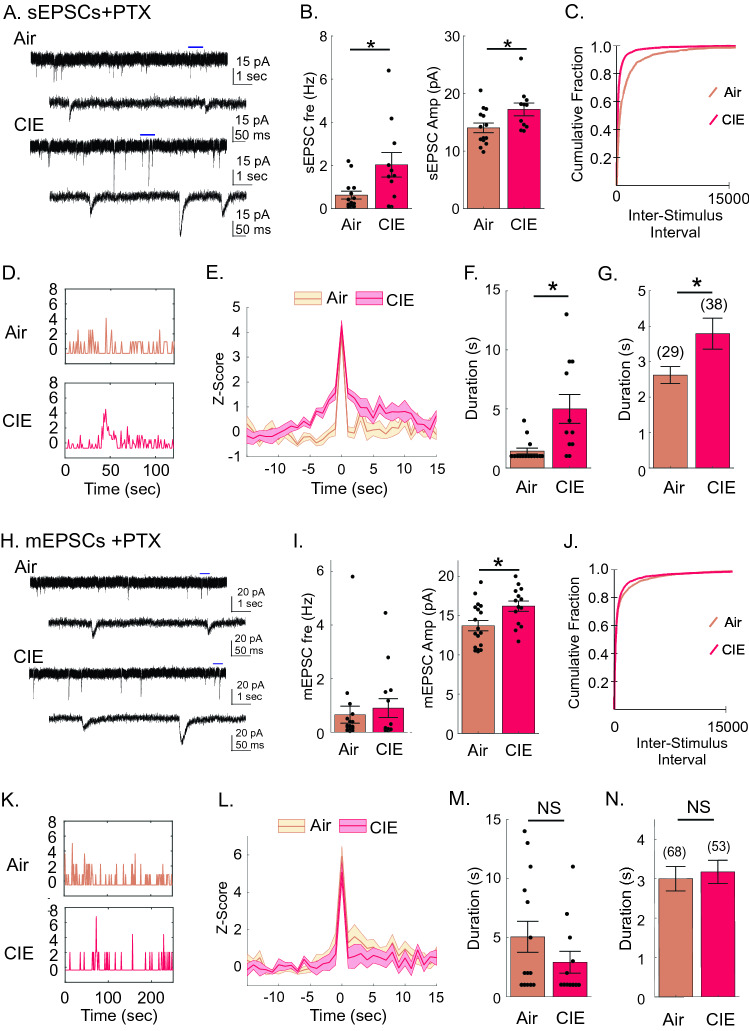
Action potential-dependent, but not action potential-independent, intrinsic network excitability is increased in CIE-treated rats. (**A**) Representative sEPSC traces recorded from vSub neurons of Air (top two traces) and CIE-treated (lower two traces) rats. (**B**) CIE rats have an increased sEPSC frequency (left graph), as well as amplitude, in the presences of PTX (right graph). (**C**) Cumulative probability plots of sEPSC inter-stimulus intervals in the population of neurons from Air and CIE-treated rats. (**D**) Representative example of an individual vSub neuron’s second-by-second z-scores (normalized sEPSC frequency) in Air (top panel) and CIE (bottom panel) rats. (**E**) Duration of z-scores aligned to the peak of z-score in Air (beige lines and shaded area, peak n = 14) and CIE-treated (red lines and shaded area, peak n = 11) rats. Lines represent mean z-score while shaded area represents SEM. (**F**) CIE-treated rats have an increased duration of peak z-scores and (**G**) duration of all instances when z-scores were above a z-score of 1. (**H**) Representative mEPSC traces recorded from vSub neurons of Air (top two traces) and CIE-treated (lower two traces) rats. (**I**) CIE rats do not show changes in mEPSC frequency (left graph), but do show an increased amplitude, in the presences of PTX (right graph). (**J**) Cumulative probability plots of mEPSC inter-stimulus intervals in the population of neurons from Air and CIE-treated rats. (**K**) Representative example of an individual vSub neuron’s second-by-second z-scores (normalized mEPSC frequency) in Air (top panel) and CIE (bottom panel) rats. (**L**) Duration of z-scores aligned to the peak of z-scores in Air (beige lines and shaded area, peak n = 14) and CIE-treated (red lines and shaded area, peak n = 12) rats. Lines represent mean z-score while shaded area represents SEM. (**M**) Air and CIE-treated rats show equivalent durations of peak z-scores and (**N**) durations of all instances when z-scores were above a z-score of 1. Numbers above bar graphs represent the number of instances when z-scores were above a z-score of 1. *p < 0.05 and errors are reported as ± SEM.

The use of optogenetics allowed us to specifically target BLA inputs to the vSub and examine the sensitivity of these synapses to CIE. An increase in di/polysynaptic activity (area) suggested a potential elevated engagement of vSub network activity in CIE-treated rats. The predominant excitatory input to the vSub comes from intrinsic projections^30^. To further examine CIE effects on intrinsic vSub synaptic activity, we recorded both spontaneous EPSCs and IPSCs of vSub neurons. Surprisingly, we found that sEPSC frequency (Air: 1.09 ± 0.26 Hz, n = 19; CIE 1.87 ± 0.32 Hz, n = 19; Mann–Whitney test, p = 0.0331), but not amplitude (Air: 15.57 ± 0.72 pA, n = 19; CIE: 17.77 ± 1.23 pA, n = 19; Mann–Whitney test, p = 0.7043), was increased in recordings from CIE rats (Fig. 5A,B). Neither the frequency (Air: 5.2 ± 1.2 Hz, n = 19; CIE 5.8 ± 0.9 Hz, n = 21; Mann–Whitney test, p = 0.3031) or amplitude (Air: 23.3 ± 2.5 pA, n = 19; CIE: 24.2 ± 2.4 pA, n = 21; Mann–Whitney test, p = 0.3462, Fig. 5C,D) of sIPSCs was altered by CIE treatment (Fig. 5C,D). The absence of a change in sEPSC amplitude, in light of an increased optically driven amplitude of BLA-specific inputs, may suggest that BLA -specific inputs are not spontaneously active and/or only contribute in a minor way to vSub sEPSCs. Additionally, the change in sEPSC frequency without a change in PPrs of optically driven events suggests that the increase in frequency was driven by an increase in intrinsic network activity and/or extrinsic inputs other than those of the BLA. To explore these possibilities further, we also studied sEPSCs under disinhibited conditions by blocking GABA_A_R-mediated inhibitory neurotransmission (using picrotoxin). Using this approach, we found a significant increase in the two cell group population responses (Air: 0.63 ± 0.18 Hz, n = 14; CIE: 2.03 ± 0.57, n = 11; Mann–Whitney test p = 0.0384, Fig. 6A,B) and this finding was confirmed by a greater cumulative fraction of shorter inter-stimulus intervals of sEPSCs from CIE rats (Kolmogorov–Smirnov test, p < 0.001, Fig. 6C). Additionally, CIE-treated rats also exhibited a higher sEPSC amplitude than their Air control counterparts (Air: 14.0 ± 0.84 Hz, n = 14; CIE: 17.2 ± 1.11, n = 11; Mann–Whitney test p = 0.0211, Fig. 6A,B). Thus, blocking network inhibition unmasked an increase in sEPSC amplitudes. A change in frequency could be driven by an increase in neuronal activity patterns (i.e. changes in action potential firing rates) within the intrinsic network neurons and/or a change in the release probability at presynaptic terminals. To dissociate between these two possibilities, we blocked both GABA_A_R-mediated inhibitory neurotransmission (PTX application) and network activity (TTX application). When we recorded the resulting mEPSCs, we found no change in the frequency of these events when comparing the two cell group population responses (Air: 0.66 ± 0.32 pA, n = 18; CIE: 0.90 ± 0.33 pA, n = 15; Mann–Whitney test p = 0.7347, Fig. 6H,I), but did detect an increase in the population inter-stimulus-interval response of CIE-treated rats (Fig. 6J). Consistent with our observation in sEPSCs with PTX, we also identified an increase in mEPSC amplitude when network inhibition was blocked (Air: 13.7 ± 0.66 pA, n = 18; CIE: 16.1 ± 0.61 pA n = 15; Mann–Whitney test p = 0.0132, Fig. 6H,I) mEPSCs in CIE rats. Taken together, these findings suggest that the intrinsic network activity is not elevated due to a change in release probability of direct contacts onto recorded vSub neurons, but rather due to an increase in the overall excitability of neurons within the network of vSub connections. We conducted a separate analysis to test this hypothesis. We performed this analysis on both sEPSCs and mEPSCs during network disinhibition. In recordings from both Air and CIE groups, we first normalized the “within neuron” frequency by comparing the frequency within each second to the mean frequency and standard deviation across the recording duration to arrive at a second by second z-score. We next found the peak z-score for each neuron and measured the duration that the neuron remained above one standard deviation of the mean frequency (z-score > 1). We observed a significant increase in the duration of maximal peak frequencies during sEPSC recordings of neurons from CIE rats (Air: 1.4 ± 0.25 s, n = 14; CIE: 5.0 ± 1.22 s, n = 11; Mann–Whitney test p = 0.0016, Fig. 6D–F) as well as an increase in the duration of all instances when frequencies were above one standard deviation of the mean frequency (z-sore > 1) (Air: 2.6 ± 0.24 s, n = 29; CIE: 3.8 ± 0.44 s n = 38; Mann–Whitney test p = 0.0314, Fig. 6D,G). When looking at the same parameters of mEPSC recordings we did not see differences in maximal peak frequency duration (Air: 5.1 ± 1.31 s, n = 14; CIE: 2.9 ± 0.92 s, n = 12; Mann–Whitney test p = 0.22200, Fig. 6K–M) or the duration of all instances when frequencies were above one standard deviation of the mean frequency (z-sore > 1) (Air: 3.0 ± 0.32 s, n = 68; CIE: 3.2 ± 0.30 s, n = 53; Mann–Whitney test p = 0.1447, Fig. 6K,N). The increase in ‘high-frequency’ duration in conjunction with an increase in frequency of spontaneous events, but no change in either parameter of miniature events, between our animal groups implicate an increase in network excitability (firing patterns) of presynaptic neurons in CIE rats.

## Discussion

In the present report, we explored the plasticity of BLA-vSub specific synapses as well as non-specific input to vSub neurons of rats during withdrawal from CIE. In BLA-vSub specific synapses we found an increase in both excitatory and inhibitory neurotransmission in vSub neurons of CIE treated rats. The overall balance in CIE rats was shifted to facilitate hyperexcitability, as evidenced by an increase in the E/I ratio and an augmentation of AMPAR-mediated conductance, driving an increase in the AMPA/NMDA ratio. All of our measures obtained in BLA-vSub specific synapses point to a postsynaptic, rather than a presynaptic form of plasticity. In contrast, when we examined spontaneous synaptic activity, likely mediated predominantly by local hippocampal circuitry, we found that CIE was associated with a significant increase in sEPSC frequency but not amplitude, arguing for an increased activity of presynaptic excitatory neurons.

Our overall findings of hyperexcitability in the vHC of CIE treated rats are consistent with a prior study from our laboratory identifying vHC hyperexcitability in CIE- treated rats on the basis of CA1 field responses^14^. The present study extends these findings to establish the subiculum of the vHC as a site of hyperexcitability. It further establishes the plasticity mechanism for a major extrinsic input (BLA input) at the single-neuron (pyramidal neuron) level. Given that activation of BLA-vHC circuitry increases anxiety-like behaviors^20,21^, the plasticity we observed at these synapses likely contributes to the negative affective state associated with withdrawal from CIE. Neuroplasticity in response to drugs of abuse in stress-sensitive circuits routinely parallels stress-induced plasticity^16,17^. In this context it is important to note that our findings of hyperexcitability in the vSub are congruent with studies of stress-induced neuroplasticity in the vHC^18,32^. As we studied the role of BLA-specific glutamatergic inputs to the vSub it is also notable that the plasticity of the BLA and its afferent inputs typically also experience a CIE-dependent increase in neuronal excitability^32–35^.

The increase in excitatory neurotransmission we established was, at least in part, driven by a functional augmentation of AMPAR-mediated currents at BLA-vSub specific synapses (mono-synaptic inputs). Support for the role of AMPAR-dependent plasticity mechanisms in driving behavioral consequences of addiction abounds^35–39^ and is also often associated with a change in the ratio of AMPA/NMDA- mediated currents^27,40^. We found an increase in AMPA/NMDA ratios at BLA-vSub synapses. Given the increase in AMPAR-mediated oEPSCs, increased functional expression of AMPAR-mediated events likely drives the increase in AMPA/NMDA ratio. As we did not pharmacologically isolate the NMDAR-mediated component, we cannot rule out NMDAR plasticity to contribute to AMPA/NMDA ratio changes. In support of a predominantly AMPAR- mediated plasticity, prior findings have reported increased expression of synaptosomal expression of the GluA2 subunit of AMPARs in the vHC, but did not find functional or molecular evidence for changes in NMDA receptor function in the dorsal or ventral hippocampus following 1 day of withdrawal from CIE^14,15^.

The increase in di/polysynaptic activity (oEPSC area) may be an extension of an increase in monosynaptic oEPSC amplitude, but based on the magnitude of difference between oEPSC areas in Air and CIE-treated rats, they likely reflect an increased engagement of local network activity. In support of this notion, we saw a substantially greater proportion of neurons with di/polysynaptic activity in CIE-treated rats (27% in Air and 79% in CIE-treated rats). In the vSub, roughly 90% of inputs are thought to arise intrinsically from the hippocampal formation with extensive connections existing between the two primary types of pyramidal neurons as well as local inhibitory neurons^30^. These data, however, do not delineate whether these increases were due purely to acutely increased BLA-driven input leading to more robust activation of connected excitatory or inhibitory neurons or whether they were also mediated by CIE-dependent plasticity of local network activity. To begin to address this question we also took measure of sEPSCs and sIPSCs, during which the contribution of the BLA to synaptic input is presumed to be low given that only BLA axons and terminal endings remain intact in the slice and the majority of vSub input arises from the hippocampus. We observed an increase in sEPSC frequency, without a change in amplitude, suggesting presynaptic but not postsynaptic plasticity in vSub neurons of CIE rats. In light of the BLA specific inputs showing post- but not pre-synaptic plasticity, this finding suggested to us that the BLA inputs did indeed not contribute substantially to sEPSC activity. Since changes in frequency suggest presynaptic adaptations, it further suggested to us that CIE can promote distinct forms of plasticity at synapses arising from extrinsic and intrinsic sources, like the BLA, and intrinsic and/or non-BLA extrinsic sources. This conclusion is supported by the fact that inhibition of action-potential dependent activity blocked the increase in the frequency of spontaneous events and that we observed an increase in the duration of time neurons of CIE treated rats spent at above average frequencies. We interpret this increase in ‘high frequency’ duration of sEPSCs, but not mEPSCs, to reflect elevated levels of network excitation that are not terminated as rapidly in CIE-treated rats. Although average mEPSC frequencies across the two treatment groups remained unchanged, we did see a small effect on the cumulative distribution of inter-stimulus intervals. We believe that this is likely due to the sensitivity of the K–S test to small differences (1 or 2 neurons with much higher frequency/shorter inter-event-intervals) that are not statistically significant across group measures. Nonetheless, this finding leaves open the possibility that changes in presynaptic release may occur in a small subpopulation of neurons and/or to a limited degree as a consequence of CIE-treatment.

When looking at sIPSCs, we saw differences between our Air and CIE-treated rats despite seeing an increase in oIPSC activity suggesting the increase is likely due or an acute di/polysynaptic inhibitory facilitation via an increase in BLA-mediated excitatory enhancement.

There are several explanations as to why the increase in BLA-vSub oEPSC amplitude was not recapitulated in our sEPSC amplitudes. In this context it is important to note that the majority (~ 90%) of inputs to vSub neurons arises from within the hippocampal formation, while the remaining ~ 10% are from extrinsic sources (including from the BLA) These values are based on anatomical measures rather than unevoked BLA specific amplitude or frequency modulation of spontaneous activity^30^. Given the relatively limited BLA-specific input to vSub neurons it is likely that BLA specific inputs have limited spontaneous activity, reducing their contribution to the overall amplitude of sEPSCs and thus limiting their detectability. Another explanation is that GABA-A receptors shunt excitatory inputs at BLA-vSub synapses (possibly other intrinsic/extrinsic inputs), to mask sEPSC amplitude changes in our CIE treated animals. There is evidence in the vSub for a role for GABA_A_R-mediated shunting inhibition of excitatory inputs^41^. Consistent with this possibility, here we show that disinhibition of network (in the presence of picrotoxin) activity unmasked an increase in sEPSC as well as mEPSC amplitude in CIE rats.

A question the two forms of plasticity in the vSub raises is whether they are linked. Does the plasticity of the BLA-vSub pathway drive the intrinsic plasticity or does the intrinsic plasticity develop as a result of other inputs to the hippocampus? Studies of CIE-dependent changes at BLA principal neurons have tracked the progression of synaptic plasticity to establish that that presynaptic plasticity precedes, but is followed by, postsynaptic plasticity^42^. If the vSub follows a plasticity progression similar to that observed in the BLA, this could imply that the postsynaptic plasticity of the BLA-vSub is a ‘more advanced’ plasticity that was preceded by changes of presynaptic release of BLA inputs. One likely scenario is that the once the BLA-dependent plasticity reaches a certain level of maturity it also facilitates changes in intrinsic and/or extrinsic network activity. Here it is important to note that the vSub does not conform to the unidirectional information flow generally associated with hippocampal connectivity. The vSub is not only heavily interconnected with itself^43^, but also makes backprojections to area CA1^43–46^ and even CA3 neurons^47^. In addition, the vSub projects back to the BLA and many other brain regions^22,48^. In particular, the vSub-nucleus accumbens (NAc) pathway plays an important role in alcohol relapse and presynaptic glutamate release is increased at vSub-NAc synapses following withdrawal from CIE^22,23,47^. The vSub-NAc circuit is also strengthened by chronic stress^49,50^ and increased vSub activity facilitates the stress response of the ventromedial hypothalamus^18^. Thus, it seems that the vSub has a central role in driving affective states and both stress and drugs of abuse can impact vSub plasticity in a similar manner. These findings raise the intriguing question of whether the vSub serves as an integral hub to consolidate affective information to form long-term memories associated with alcohol and other abused substances?

In conclusion, our findings reveal that withdrawal from chronic ethanol strengthens BLA-vSub synaptic transmission as well as action potential-dependent local network excitability. The involvement of this novel circuit in the pathophysiology of a rodent model of AUD suggests that targeting the CIE-associated hyperexcitability within this circuit may have therapeutic potential for the treatment of AUD and its comorbid conditions. Future studies will need explore the specific mechanisms that contribute to the imbalances of excitatory and inhibitory neurotransmission promoted by CIE that may identify targeted treatment approaches for the specific pathophysiological phenotoypes associated with AUD.

## Methods

### Animals

Male Long Evans rats were purchased from Envigo, IN and arrived at 250–300 g. Upon arrival rats were singly housed in clear cages (25.4 cm × 45.7 cm) and maintained on a 12:12 h light dark cycle with. Rats had ad libitum access to food (Prolab RMH 3000, LabDiet: PMI Nutrition International, St. Louis, MO) and water throughout the study. Animal care procedures were carried out in accordance with the NIH Guide for the Care and Use of Laboratory Animals and were approved by the Wake Forest University Animal Care and Use Committee. All animal care and use procedures were carried out in compliance with ARRIVE guidelines. A total of 15 CIE and 16 AIR rats were used to complete all electrophysiological studies.

### Stereotaxic surgeries

Naïve subjects were anesthetized using sodium pentobarbitol (40–60 mg/kg, i.p.). The scalp was shaved and surgically scrubbed. Rats were placed in a stereotaxic frame and an incision was made centrally along the scalp. A craniotomy positioned directly above the posterior BLA (stereotaxic coordinates in mm were AP:-3.45 ML: 5.0 and DV:7.0) was made bilaterally to allow microinjection needles to be lowered into the pBLA. Microinjection needles were used to transfect the pBLA with 0.8 μL of a virus construct (pAAV5-CaMKIIa-hChR2(H134R)-EYFP) expressing the excitatory opsin, channelrhodopsin (ChR2) at a rate of 2 μL/min (Addgene, Cambridge, MA). Microinjection needles were maintained in place for 5–7 min before being withdrawn. Animals were sutured and allowed to recover in their home cages. The viral construct was allowed to express and traffic to BLA terminals for the following 5 weeks and subsequently exposed to 10–12 days of chronic intermittent ethanol vapor inhalation or air exposure.

### Chronic intermittent ethanol vapor inhalation exposure

Animals in the CIE condition were housed in their standard home cages which were placed in custom-built Plexiglas chambers (Triad Plastics, Winston-Salem, NC). Ethanol vapor was pumped into the chamber for 12 h a day for 10–12 consecutive days during the light cycle (reverse light cycle 9 p.m. to 9 a.m.). This regimen was selected because it led to a significant increase in anxiety-like behaviors in this strain of rats in a prior study., Control animals (air) were similarly housed in a reverse light cycle but were exposed only to room air. Animals were weighed daily and tail blood samples were taken a minimum of 3 times during the 10–12 days of CIE procedure at 9 a.m. to monitor blood ethanol concentrations (BECs). Following the 10–12 days of CIE, animals underwent 24 h of withdrawal (no ethanol vapor) and were sacrificed for electrophysiological recordings^14^.

### Blood ethanol determination

Blood ethanol concentrations (BECs) were determined from a 10 μL sample of tail vein blood obtained via tail snip from each individual rat. BECs were determined using a commercially available alcohol dehydrogenase enzymatic assay kit (Carolina Liquid Chemistries Corporation, Brea, CA). Ethanol concentrations were then determined using a spectrophotometer (Molecular Devices Spectra Max). Only animals that maintained an average BEC between 150 and 250 mg/dL across all measurements were included in the analysis in the results presented (results of 1 animal had to be excluded due to a significantly lower BEC average). The average BEC of all included animals was 193.8 ± 9.2 mg/dL^14^.

### Electrophysiology

Electrophysiological experiments were conducted following 24 h of withdrawal from CIE. Animals were deeply anesthetized using isoflurane. Following decapitation, the brain was removed rapidly and suspended in ice-cold NMDG recovery solution containing in mM: 92 NMDG, 2.5 KCl, 1.25 NaH_2_PO_4_, 30 NaHCO_3_, 20 HEPES, 25 glucose, 2 thiourea, 5 Na-ascorbate, 3 Na-pyruvate, 0.5 CaCl_2_·2H_2_O, and 10 MgSO_4_·7H_2_O. NMDG was titrated to pH 7.4 with 17 mL ± 0.5 mL of 5 M hydrochloric acid^51^. Transverse slices containing the vHC were cut at a thickness of 300 μm using a VT1000S Vibratome (Leica Microsystems). vHC slices were placed in a holding chamber containing NMDG recovery solution. Slices were allowed to recover for 35 min before being transferred to a chamber filled with artificial cerebral spinal fluid (aCSF) containing in mM: 125 NaCl, 1.25 NaH_2_PO_4_, 25 NaHCO_3_, 10 D-Glucose, 2.5 KCl, 1 MgCl_2_, and 2 CaCl_2_. NMDG recovery and aCSF holding solutions were oxygenated with 95% O_2_ and 5% and warmed to 32–34 °C^14^. For recordings, a single brain slice was transferred to a chamber mounted on a fixed stage under an upright microscope (Scientifica SliceScope Pro 2000 microscope), where it was superfused continuously with warmed oxygenated aCSF. Whole-cell voltage-clamp recordings were made in presumptive pyramidal neurons of the ventral subiculum (vSub) using recording pipettes pulled from borosilicate glass (open tip resistance of 6–9 MΩ; King Precision Glass Co., Claremont, CA). The pipette solution contained (in mM): 130–140 Cs-gluconate, 10 HEPES, 1 NaCl, 1 CaCl_2_, 3 CsOH, 5 EGTA, 2 Mg^2+^-ATP, 0.3 GTP-Na_2_ and 2 Qx-314. Intracellular Cs^+^ was used as the primary cation carrier in voltage-clamp recordings to block K^+^ currents, including postsynaptic GABA_B_ receptor-mediated currents, in the recorded neuron^29^.

Neurons in the ventral subiculum (vSub) were targeted for recording under a 40 × water-immersion objective (numerical aperture = 0.8) with infrared-differential interference contrast (IR-DIC) optics, as described previously. Electrophysiological signals were obtained using a Multiclamp 700B amplifier (Molecular Devices, Union City, CA), low-pass filtered at 2 or 3 kHz, digitized at 10 kHz, and recorded onto a computer (Digidata 1440A, Molecular Devices) using pClamp 11.0 software (Molecular Devices). Seal resistance was typically 2–5 GΩ and series resistance, measured from brief voltage steps applied through the recording pipette (5 mV, 5 ms), was < 25 MΩ and was monitored periodically during the recording. Recordings were discarded if series resistance changed by > 20% over the course of the experiment. Each recorded neuron represented an individual data point (n); recordings were made from at least five rats for each experimental group. Optical stimulation was performed using blue light (473 nm) to stimulate terminals synapsing onto vSub neurons originating from the pBLA. For recordings of optically- evoked excitatory and inhibitory postsynaptic currents (oEPSCs and oIPSCs, respectively), cells were voltage clamped at − 70 mV (near the theoretical IPSC reversal potential) and at 0 mV (near the theoretical EPSC reversal potential), respectively. To establish maximal monosynaptic amplitudes and polysynaptic areas under the curve cells, were stimulated for 5 ms and at 34 mW/mm^2^ intensity with the cell centered over the light source. Optical stimulation was performed at 0.1 Hz to obtain at least 10 consecutive optically- evoked postsynaptic currents. To establish the excitatory and inhibitory ratio (E/I ratio), the total excitatory area was divided by the total inhibitory area when both could be obtained from the same neuron. A minimum of 7 amplitude samples were averaged to arrive at an average response amplitude of presumptive monosynaptic oEPSCs. oEPSCs. Paired-pulse ratios (PPrs) were determined by delivering paired optical stimulations at an interstimulus interval (ISI) of 100 or 250 ms. Stimulation duration and intensity for PPr recordings were adjusted to obtain monosynaptic responses. PPrs were obtained by taking the ratio of the second and first amplitude (Amp2/Amp1). For recordings isolating α-amino-3-hydroxy-5-methyl-4-isoxazolepropionic acid (AMPA) receptor mediated oEPSCs cells and to establish the amplitude of NMDA-receptor- mediated oEPSCs cells were voltage-clamped at − 80 and + 40 mV, respectively. To determine the AMPA/NMDA ratio, stimulation duration and intensities were adjusted to obtain a minimum of 7 monosynaptic oEPSCs^52^. The maximum NMDA- mediated amplitude was determined at the time of completion of the AMPA mediated oEPSCs. The AMPA/NMDA ratio was established as the ratio between the AMPA and NMDA mediated amplitudes. To establish the duration of peak frequencies and the duration of frequencies that were above one standard deviation of the mean (z-score > 1), frequency measures for each neuron were first normalized using z-scores. The z-score at each recorded second was determined according to $z=\frac{x-\mu }{\sigma }$ where x is the average frequency over each one second interval, µ is the average frequency across the entire recording duration and σ is the standard deviation of frequency across the entire recording duration. For determining the normalized peak frequency duration, the total duration for which averaged one second frequencies remained above a z-score of 1 surrounding maximum z-scores was determined. To establish all instances of above average frequencies, we determined the duration across all instances when frequencies were above a z-score of 1. We counted only instances where frequencies were above z-score of one for at least 2 consecutive seconds.

### Drug application

For a subset of experiments several pharmacological reagents were bath applied. Picrotoxin was bath applied to block GABA_A_ receptors to pharmacologically isolate oEPSCs for AMPA receptor- mediated oEPSCs and AMPA/NMDA ratios. Recordings in the presence of Tetrodotoxin (TTX; 1–2 μM; Tocris Bioscience, Minneapolis, MN) were made to record action potential-independent (i.e., miniature) excitatory postsynaptic currents (i.e., mEPSCs) or to inhibit optically evoked activity. 4-Aminopyradine (500 μM) was added in combination with TTX to unmask optically evoked activity blocked by TTX. Picrotoxin (100 μM; Sigma-Aldrich, St. Louis, MO) was added to the ACSF to block GABA_A_ receptors. For specific experiments, DL-2-Amino-5-phosphonopentanoic acid (AP-5; 100 μM), NMDA (300 μM), and 6,7-dinitroquinoxaline-2,3-dione (DNQX; 20 μM; all from Sigma-Aldrich) was added to ACSF to block AMPA receptor mediated conductance.

### Statistical analysis

Statistical analysis was performed using MiniAnlaysis, Matlab and Prism. The normality of measures (amplitudes, areas, PPrs and ratios) was tested using a Shapiro–Wilk test. A repeated measures ANOVA was used to test the effect of multiple drug treatments of oEPSC. Significant differences between the means of Air and CIE measures were assessed using a two-tailed unpaired student’s t-test or a Mann–Whitney test. All results were considered statistically significant with a p < 0.05. Errors are reported as ± SEM.
